# Nutritional status and gaps in nutritional care among adolescents living with HIV in Ethiopia: A multi-facility study

**DOI:** 10.1371/journal.pgph.0004995

**Published:** 2026-07-08

**Authors:** Meless Gebrie Bore, Lin Perry, Xiaoyue Xu, Andargachew Kassa Biratu, Marilyn Cruickshank

**Affiliations:** 1 College of Medicine and Health Science, Hawassa University, Hawassa, Ethiopia; 2 Faculty of Health, School of Nursing and Midwifery, University of Technology Sydney, Ultimo, New South Wales, Australia; 3 Faculty of Medicine, School of Population Health, University of New South Wales, Ultimo, New South Wales, Australia; 4 Sydney Children’s Hospital Network, Sydney, New South Wales, Australia; PLOS: Public Library of Science, UNITED STATES OF AMERICA

## Abstract

Adolescents living with HIV (ALHIV) in Ethiopia face significant nutritional challenges due to increased energy requirements, HIV-related complications, and socioeconomic constraints. This study assessed the nutritional status of ALHIV, examined associated factors, and evaluated gaps in the provision of nutritional care within antiretroviral therapy (ART) clinics. Two cross-sectional surveys were conducted in ten public hospitals across Addis Ababa and Oromia, involving 384 ALHIV aged 10–19 years and 44 healthcare professionals. ALHIV were selected through proportionate random sampling, while healthcare workers were purposively recruited. Data were collected using a pre-tested, structured questionnaire with both quantitative and qualitative components, administered by trained staff via the Kobo-Toolbox. Quantitative data were analyzed using descriptive statistics and logistic regression, while qualitative responses were analyzed using thematic analysis. The study included adolescents with a mean age of 15.9 years, of whom 54% were female. Overall, 24.2% of ALHIV were thin, 21.7% were stunted, and 34.9% were malnourished, including 19.4% with severe acute malnutrition. Food insecurity was a key factor negatively affecting nutrition and ART adherence. Although routine anthropometric assessments were commonly performed, only 36.4% of healthcare providers assessed dietary intake, 27.3% evaluated food security, and 38.6% provided regular nutrition counseling. Healthcare providers reported barriers including limited training, heavy workload, and resource constraints. Malnutrition remains a significant concern among ALHIV in Ethiopia, while several components of recommended nutritional care are inconsistently implemented in ART clinics. Strengthening the integration of comprehensive nutrition services, including routine dietary assessment, nutrition counseling, and food security screening, into ART care programs may improve the health and treatment outcomes of ALHIV.

## 1. Introduction

Adolescents living with HIV (ALHIV) face a dual burden of managing a chronic infection during a life stage characterized by rapid physical, emotional, and cognitive development [1]. In this context, the dual burden refers to the coexistence of undernutrition and emerging forms of malnutrition, such as overweight and diet-related non-communicable diseases, among adolescents affected by HIV. In Ethiopia, where adolescents represent approximately 33% of the population [2], this large demographic group constitutes a critical population for targeted health and nutrition interventions. Adolescents living with HIV are particularly vulnerable to malnutrition due to increased nutritional needs, the physiological impacts of HIV, and widespread food insecurity. Malnutrition among ALHIV undermines immune function, increases susceptibility to infections, and impairs treatment outcomes, presenting a critical challenge to adolescent health and HIV care systems [3].

Adequate nutrition is essential for optimal antiretroviral therapy (ART) effectiveness and long-term health outcomes among adolescents [4,5]. HIV further elevates energy and nutrient requirements, and poor nutritional status in adolescence can impair growth, delay puberty, and increase the risk of non-communicable diseases in adulthood [5–7]. Studies have documented that ALHIV commonly experience undernutrition, micronutrient deficiencies, and, in some contexts, overweight or obesity [3]. In Ethiopia, these challenges are intensified by food insecurity, limited healthcare infrastructure, and inadequate integration of nutritional services into ART care [8,9]. Factors such as peer pressure, household income, parental influence, and cultural beliefs also complicate adolescent dietary behaviors and adherence to nutritional guidance [10]. Despite national guidelines on HIV and adolescent nutrition, there is limited empirical evidence from Ethiopia on how nutritional assessment, counselling, and support are operationalized within ART clinic for adolescents.

While the role of nutrition in the management of HIV is well-established, there is limited evidence from Ethiopia on the actual nutritional status of ALHIV and the practices of healthcare providers in assessing and addressing their nutritional needs. Specifically, few studies have examined how healthcare workers assess nutritional status, provide counselling, or implement support services in ART settings. Moreover, there is a lack of data on the tools, training, and resources available to healthcare workers to deliver comprehensive nutritional care for ALHIV.

This study aimed primarily to assess the anthropometric and nutritional status of adolescents receiving ART in Ethiopia. In addition, the study sought to explore nutritional assessment, counselling, and support practices of healthcare professionals working in ART clinics. By identifying gaps in service delivery and highlighting areas for improvement, the study ultimately aims to inform evidence-based strategies and policies to strengthen nutritional care and improve health outcomes for adolescents living with HIV.

## 2. Methods

### 2.1. Study setting and period

This study was conducted between September 2023 and February 2024 across two regions in Ethiopia: Addis Ababa and Oromia. Ten hospitals were purposively selected based on their high HIV burden and large number of ART recipients: Adama Referral Hospital, ALERT General Hospital, Asella Referral and Teaching Hospital, Batu Hospital, Bishoftu Hospital, Ras Desta Damtew Hospital, Shashamene Comprehensive Specialized Hospital, St Paul’s Comprehensive Specialized Hospital, Yekatit 12 Hospital, and Zewditu Hospital. These facilities are major public hospitals providing comprehensive HIV care and antiretroviral therapy (ART) service in Ethiopia’s urban and peri-urban settings [11].

### 2.2. Study design and population

We conducted an institution-based cross-sectional study using a mixed-methods sequential explanatory design that integrated quantitative and qualitative components. The quantitative survey assessed the nutritional status of ALHIV and evaluated healthcare workers’ practices related to nutritional assessment, counselling, and management. Qualitative responses from healthcare professionals were collected to provide contextual insight into barriers and challenges affecting the delivery of nutritional care in ART clinics. The study adhered to the Strengthening the Reporting of Observational Studies in Epidemiology – Nutritional Epidemiology (STROBE-Nut) guidelines [12–14].

### 2.3. Sampling and recruitment

#### 2.3.1. Sampling of ALHIV.

We used a proportionate random sampling strategy to recruit ALHIV aged 10–19 years based on data provided by ART data clerks at each facility. Recruitment of ALHIV took place between September 2023 and February 2024. We developed a sampling frame using ART registration codes from each facility. Participants were assigned unique research codes to ensure confidentiality.

Sample sizes were allocated proportionally according to the number of ALHIV in each hospital. Within each hospital, we randomly selected eligible participants using SPSS version 26 based on their registration numbers.

**ALHIV Inclusion and Exclusion Criteria:**

Adolescents living with HIV (ALHIV) were eligible for inclusion if they were aged 10–19 years, currently receiving antiretroviral therapy (ART), and receiving care at one of the selected hospitals. Participants were excluded if they had been on ART for less than three months, had cognitive or communication impairments that could hinder reliable participation, were under 18 years of age without guardian or parental consent, or lacked an ART registration number or verifiable identity.

#### 2.3.2. Sampling of healthcare professionals.

We used a purposive sampling method to recruit healthcare professionals directly involved in providing ART services to adolescents living with HIV at each study site. These participants provided qualitative insights into their practices in nutritional assessment and support. The purposive approach allowed inclusion of professionals with direct experience in adolescent HIV care and nutritional management.

**Inclusion and Exclusion Criteria:**

Healthcare workers were eligible for inclusion if they were actively involved in delivering antiretroviral therapy (ART) to adolescents living with HIV (ALHIV) at the selected facilities. Healthcare workers were excluded if they had been employed in the ART unit for less than three months.

### 2.4. Sample size determination

#### 2.4.1. ALHIV sample size.

The sample size for ALHIV was determined based on three primary objectives: assessing nutritional status, identifying influencing factors, and evaluating food consumption patterns. The largest required sample size across these objectives was selected to ensure adequate power.

**Nutritional status:** Using a single population proportion formula (5% margin of error, 95% CI, and a 33.1% undernutrition prevalence from a 2020 study in southern Ethiopia [15]), the required sample size was 340 participants.
**Influencing factors:** A two-population proportion formula (5% type I error, 80% power, 1:1 ratio) was used to calculate a sample size of 352 participants for the variable “meal skipping” [15–17].
**Food consumption patterns:** Assuming a 50% prevalence (due to lack of prior data), the estimated sample size was 384 participants.

The final sample size of 384 ALHIV was selected to satisfy all study objectives.

#### 2.4.2. Healthcare professional sample size.

A total of 50 healthcare professionals were identified across the ten hospitals (approximately five per site). Of these, four were unavailable due to annual leave (n = 1), offsite training (n = 2), or maternity leave (n = 1). Among the 46 invited participants, 44 consented to participate.

### 2.5. Data collection

Data were collected using a pre-tested, interviewer-administered structured questionnaire, which included both closed- and open-ended questions tailored for ALHIV and healthcare professionals. The Kobo Toolbox platform [18] was used to facilitate electronic data collection via smartphones and computers. Trained data collectors conducted the interviews after receiving standardized training in data collection procedures and ethical research conduct.

A three-day training workshop was held prior to data collection to prepare the data collectors. The training covered the study objectives, data collection tools, interview techniques, and procedures for nutritional assessments, including anthropometric measurements, clinical examinations, and dietary intake assessments.

Clinical assessments followed standardized protocols (see S1 Text) to ensure consistency and accuracy. Measurements were performed using calibrated instruments, including digital scales and portable stadiometers [19]. Physical examinations assessed clinical signs of malnutrition, including wasting, bilateral pitting oedema, palmar pallor, hair changes, and muscle loss.

To verify the reliability of anthropometric measurements, the Intraclass Correlation Coefficient (ICC) was calculated, yielding a value of 0.856, indicating good measurement agreement [20,21].

### 2.6. Survey questionnaires

The questionnaires were initially developed in English and translated into Amharic. To ensure linguistic accuracy, the Amharic version was back-translated into English by a language expert, and both versions were compared for consistency [22].

Content validity was assessed using the Item-Level Content Validity Index (I-CVI) and the Scale-Level Content Validity Index (S-CVI) [23,24], with a threshold of 0.78 or higher indicating acceptable relevance [25].

Inter-rater consistency was evaluated using Cohen’s Kappa coefficient, which achieved a value of 0.71, indicating substantial agreement [20]. Qualitative feedback from the expert panel was incorporated to improve clarity and refine the questionnaire.

### 2.7. Data processing and analysis

All quantitative data were analyzed using SPSS version 21. Descriptive statistics were generated to examine nutrition-related assessment, counselling, and management practices. Frequencies, percentages, and measures of central tendency were calculated, and the results were presented in tables and figures. The datasets were screened for outliers and inconsistencies prior to analysis.

BMI-for-age Z-score (BAZ) was selected as the primary outcome variable due to its clinical relevance and routine use for nutritional monitoring among adolescents receiving ART. Other anthropometric indicators, including height-for-age Z-score (HAZ) and mid-upper arm circumference (MUAC), were used to assess stunting and acute malnutrition respectively.

Normality of continuous variables was assessed using both the Kolmogorov-Smirnov and Shapiro-Wilk tests (significance threshold p < 0.05) and visually validated through Q-Q plots. The Shapiro-Wilk test was used for its greater statistical power with smaller samples, while the Kolmogorov-Smirnov test was used as an additional verification method. To identify associations among anthropometric indices, correlational and linear regression analyses were conducted.

Open-ended qualitative responses from healthcare professionals were analyzed using a descriptive thematic approach to contextualize findings and identify implementation barriers. Two researchers independently reviewed and coded responses, then grouped them into thematic categories. These themes were then integrated with the quantitative findings using a mixed-methods triangulation approach to provide deeper insights into healthcare professionals’ practices. Participants were identified by study codes to maintain confidentiality.

Operational definitions used in the study are provided in Supportive Information (S2 Text).

### 2.8. Ethics approval

This study was conducted in accordance with the Declaration of Helsinki. Ethical approval was obtained from the Human Research Ethics Committee, University of Technology Sydney, Australia (Ref.: ETH23–7873); the Institutional Review Board of the College of Medicine and Health Sciences, Hawassa University, Ethiopia (Ref.: IRB/321/15); the regional Health Bureau Ethics Review Committee (Ref: A/A/3/54/227); and the Ethics Committee of the participating hospitals. Permission was obtained from all participating health institutions prior to data collection.

Written informed consent was obtained from participants aged 18–19 years and from parents or legal guardians of participants aged under 18 years, with assent obtained from adolescents where appropriate. Participant confidentiality and privacy were strictly maintained throughout the study, and all data were de-identified prior to analysis.

## 3. Results

### 3.1. Participants’ characteristics

#### 3.1.1. Adolescents living with HIV (ALHIV).

A total of 384 adolescents living with HIV (ALHIV) receiving ART follow-up services participated in the study, yielding a 100% response rate. The mean age of participants was 15.9 ± 2.19 years, with the majority (n = 227; 59.1%) aged between 14 and 17 years, representing the middle adolescent age group. More than half were female (n = 207; 54%). Nearly all participants were students (n = 379; 98.7%), with close to half (n = 179; 46.6%) enrolled in grades 1–8.

In terms of household characteristics, the largest proportion of participants came from households earning between 1,000 and 3,000 Ethiopian Birr (EBR) per month (n = 175; 45.6%). More than half (n = 198; 51.6%) lived in households with 4–5 members (Table 1a).

**Table 1:** (a) Socio-demographic characteristics of 384 ALHIV attending ART follow-up across 10 selected hospitals in Addis Ababa and Oromia regions, Ethiopia. (b) Socio-Demographic Characteristics of the ART Clinic Healthcare Workers in the Selected Hospitals of two Regions of Ethiopia, 2024.

(a) Socio-demographic characteristics of 384 ALHIV attending ART follow-up across 10 selected hospitals in Addis Ababa and Oromia regions, Ethiopia		
Variables	Description	Frequency n (%)
Age (years)	10–13 years (early adolescent)	55 (14.3)
	14–17 years (mid-age adolescent)	227 (59.1)
	18–19 years (late adolescent)	102 (26.6)
Mean age ± SD	15.9 ± 2.19 years	
Sex	Male	177 (46.1)
	Female	207 (53.9)
Region	Addis Ababa	254 (66.1)
	Oromia	130 (33.9)
Religion	Orthodox	281 (73.2)
	Muslim	49 (12.8)
	Protestant	48 (12.5)
	Others *	6 (1.5)
Highest educational grade	Grade 1–8	179 (46.6)
	Grade 9–10	111(28.9)
	Grade 11 and above	94 (24.5)
Occupation	Student	379 (98.7)
	Daily laborer	3 (0.8)
	Others (industry employees, begging)	2 (0.5)
Family monthly income	<1000 EBR	41(10.7)
	1000– < 3000 EBR	175 (45.6)
	≥3000–5000 EBR	94 (24.5)
	≥ 5000 EBR	74 (19.3)
Family size	< 4	130 (33.9)
	4 – 5	198 (51.6)
	≥ 6	56 (14.6)
Living situation	Living with parents/responsible adult	369 (96.1)
	Living alone or with peers	15(3.9)
(b) Socio-Demographic Characteristics of the ART Clinic Healthcare Workers in the Selected Hospitals of two Regions of Ethiopia, 2024		
**Variables**	**Description**	**Frequency N (%)**
Age (year)	24 – 34 years	21(47.7)
	35 – 44 years	16 (36.4)
	≥ 45 years	7 (15.9)
	Mean ± SD = 35.4 ± 6.99 year	
Sex	Male	17 (38.6)
	Female	27 (61.4)
Region	Addis Ababa	25 (56.8)
	Oromia	19 (43.2)
Educational Status	Diploma Graduate	4 (9.1)
	Bachelor’s Degree Graduate	34 (77.3)
	Master’s Degree Graduate	6 (13.6)
Profession	Registered Nurse	28 (63.6)
	Public Health Officer	6 (13.6)
	General Practitioner	10 (22.7)
Length of Work Experience	< 10 years	26 (59.1)
	10 – 20 years	14 (31.8)
	≥ 21 years	4 (9.1)
Mean ± SD =	10.7 ± 6.61 year	
Length of Work Experience in ART unit/clinic	< 10 years	36 (81.9)
	≥ 10 years	8 (18.2)
Mean ± SD =	5.45 ± 4.23 year	
Monthly Income	5000 – 7500 EBR	9 (20.5)
	7501 – 10000 EBR	19 (43.2)
	> 10000 EBR	16 (36.4)

Others* - Catholic, Jehovah’s Witness; SD = Standard Deviation; EBR = Ethiopian Birr.

SD - Standard Deviation.

#### 3.1.2. Healthcare professionals.

A total of 44 healthcare workers participated, reflecting a 95.6% response rate. The mean age of respondents was 35.4 ± 6.99 years, with nearly half (n = 21; 47.7%) aged 24–34 years. More than half were recruited from Addis Ababa regional hospitals (n = 25; 56.8%).

In terms of professional background, most respondents held bachelor’s degrees (n = 34; 77.3%) and were Registered Nurses (n = 28; 63.6%). The majority (n = 26; 59.1%) had less than 10 years of professional experience, and most reported previous work in an ART clinic (n = 36; 81.9%) (Table 1b).

### 3.2. Anthropometric and nutritional status of adolescents living with HIV

#### 3.2.1. Anthropometric measurements.

The mean height and weight of ALHIV participants were 155.7 ± 10.3 cm and 44.5 ± 8.6 kg, respectively. The mean body mass index (BMI) was 16.8 ± 2.5 kg/m^2^, and the mean mid-upper arm circumference (MUAC) was 21.0 ± 2.8 cm. Statistically significant differences in anthropometric parameters were observed across different age groups (Table 2).

**Table 2:** Descriptive analysis of ALHIVs’ anthropometric variables by age category.

Description	Mean ± SD by age (years)				Test result (F-statistics value, df, p-value)
	10–13	14–17	18–19	10–19	
	(n = 55)	(n = 227)	(n = 102)	(n = 384)	
Height (cm)	143.3 ± 8.6	157.2 ± 9.1	159.3 ± 8.9	155.7 ± 10.3	F = 64.3, df = 2, P < 0.001
Weight (kg)	34.8 ± 7.1	44.7 ± 7.2	49.0 ± 8.2	44.5 ± 8.6	F = 65.6, df = 2, p < 0.001
BMI (kg/m^2^)	16.8 ± 2.5	18.1 ± 2.7	19.4 ± 3.2	18.3 ± 2.9	F = 15.2, df = 2, p < 0.001
MUAC (cm)	18.8 ± 1.9	21.0 ± 2.5	22.4 ± 2.9	21.1 ± 2.8	F = 37.3, df = 2, p < 0.001
Skinfold thickness					
Biceps (mm)	4.35 ± 2.0	4.85 ± 2.16	4.93 ± 2.7	4.8 ± 2.3	F = 1.29, df = 2, p = 0.278
Triceps (mm)	6.13 ± 2.5	8.0 ± 4.1	8.24 ± 4.5	7.8 ± 4.1	F = 5.75, df = 2, p = 0.003
Subscapular (mm)	6.53 ± 2.7	8.9 ± 4.6	10.2 ± 6.6	8.9 ± 5.1	F = 9.70, df = 2, p < 0.001
Supra-iliac (mm)	4.9 ± 2.3	7.4 ± 3.9	8.3 ± 4.6	7.3 ± 4.0	F = 13.5, df = 2, p < 0.001
S_4_SKT (mm).	21.9 ± 8.5	29.2 ± 13.1	31.7 ± 16.1	28.8 ± 13.7	F = 9.66, df = 2, p < 0.001
Waist circumference (cm)	56.1 ± 7.6	64.4 ± 6.6	66.8 ± 7.3	63.8 ± 7.7	F = 44.2, df = 2, P < 0.001
Hip circumference (cm)	68.8 ± 8.8	81.5 ± 7.6	84.9 ± 7.9	80.6 ± 9.3	F = 79.7, df = 2, p < 0.001
Waist-to-hip ratio	0.82 ± 0.05	0.79 ± 0.06	0.78 ± 0.1	0.79 ± 0.1	F = 3.86, df = 2, p = 0.022
Waist-to-height ratio	0.39 ± 0.05	0.41 ± 0.04	0.42 ± 0.1	0.41 ± 0.5	F = 6.1, df = 2, p = 0.002
Body fat percentage	20.3 ± 10.5	18.9 ± 8.4	19.5 ± 8.6	19.3 ± 8.7	F = 9.66, df = 2, p = 0.526
Grip strength					
Left hand (kg)	15.5 ± 4.8	21.3 ± 6.7	23.1 ± 8.0	20.9 ± 7.2	F = 22.5, df = 2, p < 0.001
Right hand (kg)	13.7 ± 4.5	19.6 ± 6.3	21.1 ± 7.8	19.1 ± 6.9	F = 24.5, df = 2, p < 0.001

ANOVA was used to compare anthropometric measurements of participants. *p ≤ 0.05, **p ≤ 0.01 and *** p ≤ 0.001. BMI = body mass index, MUAC – mid-upper arm circumference, S_4_SKT = sum of skinfold thickness.

#### 3.2.2. Nutritional status.

Nutritional status was assessed using three indicators: BMI-for-Age Z-score (BAZ) to determine thinness, Height-for-Age Z-score (HAZ) for stunting, and MUAC-for-age for acute malnutrition.

**a) Thinness Based on BMI-for-Age**

**Fig 1:**
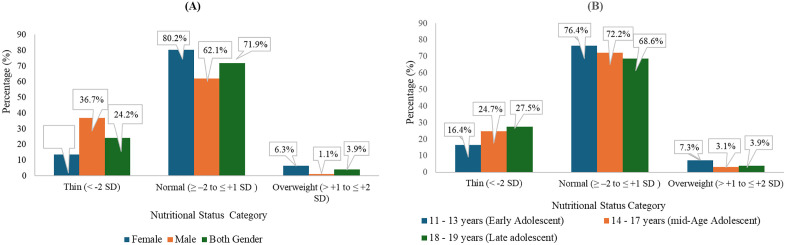
Nutritional status based on BMI-for-age among adolescents living with HIV (ALHIV) on ART follow-up. **(A)** Level of undernutrition according to gender. **(B)** Distribution of nutritional status according to age category.

Using the 2007 WHO Growth Reference [26], 24.2% (n = 93) of ALHIV were classified as thin (BAZ < -2 SD), of whom 26.9% (n = 25) were severely thin (BAZ < -3 SD) and 73.1% (n = 68) moderately thin (-3 SD ≤ BAZ < -2 SD) [26–28](Fig 1A). Thinness was more prevalent among males than females. The highest prevalence of thinness was observed in late adolescence (Fig 1B).

**b) Stunting Based on Height-for-Age**

**Fig 2:**
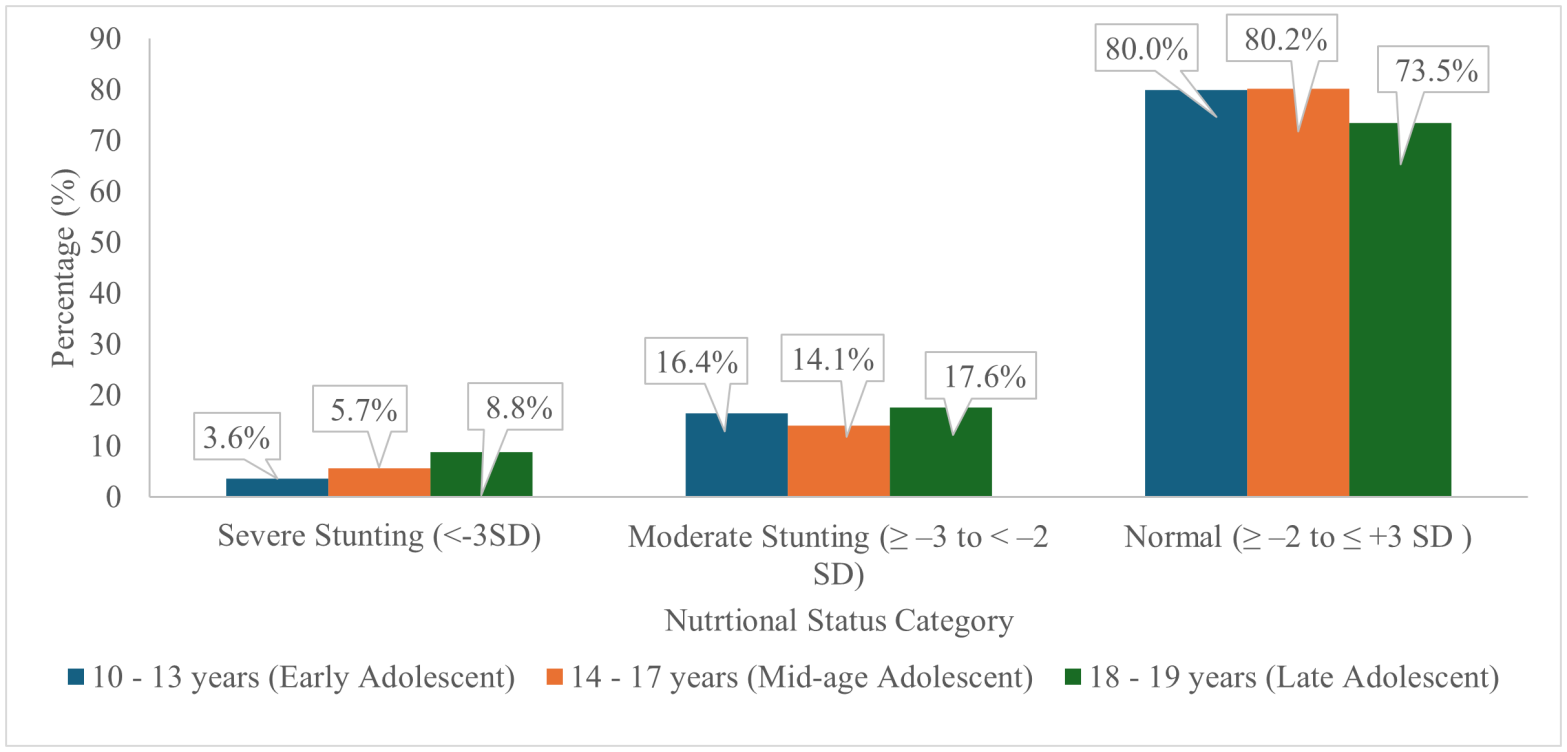
Distribution of stunting status according to age category among adolescents living with HIV (ALHIV) on ART follow-up.

A total of 21.7% (n = 83) of participants were stunted (HAZ < -2 SD), with 28.9% (n = 24) of these classified as severely stunted (HAZ < -3 SD) [26]. Severe stunting was most common among late adolescents (8.8%). Stunting prevalence was slightly higher in males (22.6%) compared to females (20.8%), with severe stunting in 7.3% of males and 5.3% of females (Fig 2).

**c) Acute Malnutrition Based on MUAC-for-Age**

Based on MUAC-for-age criteria, 34.9% (n = 134) of participants were malnourished [29], including 80.6% (n = 108) with moderate acute malnutrition and 19.4% (n = 26) with severe acute malnutrition.

**Fig 3:**
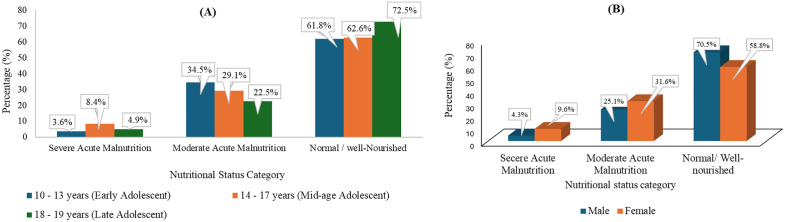
Malnutrition status based on MUAC measurements among adolescents living with HIV (ALHIV) on ART follow-up. **(A)** Distribution according to age category. **(B)** Distribution according to gender.

Mid-adolescents (ages 14–17) had the highest prevalence of acute malnutrition (37.4%, n = 85), including 8.4% (n = 19) with severe acute malnutrition. In comparison, the early adolescent group had a lower prevalence of severe acute malnutrition (3.6%, n = 2), as did late adolescents (4.9%, n = 5). Moderate acute malnutrition was most prevalent among early adolescents (34.5%) (Fig 3A).

Male participants were more likely to be acutely malnourished (41.2%, n = 73) compared to females (29.4%, n = 61). Severe acute malnutrition was recorded in 9.6% of males (n = 17) versus 4.3% of females (n = 9) (Fig 3B).

### 3.3. Anthropometric parameters associated with malnutrition

#### 3.3.1. Correlation analysis.

Moderate positive correlations were found between the sum of four skinfold thickness measurements and both body fat percentage (r = 0.436, p < 0.001) and BMI-for-age (r = 0.335, p < 0.001), suggesting that increases in skinfold thickness are associated with higher adiposity and BMI. The correlation between skinfold thickness and MUAC-for-age was weaker (r = 0.296, p < 0.001), indicating that MUAC may reflect muscle mass and other components not directly captured by skinfold measures (Table 3).

**Table 3:** Pearson correlation analysis for anthropometric indices.

Variables	BFP	BMI-for-Age (BAZ)	MUAC-for-age (cm)	S_4_SKT (mm).
BFP	–	0.264^**^	0.148^**^	0.436^**^
BMI-for-Age (BAZ)	0.264^**^	–	0.377^**^	0.335^**^
MUAC-for-age	0.148^**^	0.377^**^	–	0.298^**^
S_4_SKT	0.436^**^	0.335^**^	0.298^**^	–

Data presented are correlation coefficient (r) values.

Bold values indicate significant positive correlations.

** p < 0.001 (2-tailed).

BFP = body fat percentage; BMI = body mass index; MUAC = mid-upper arm circumference; S_4_SKT = the sum of four skinfold thicknesses (biceps, triceps, subscapular, and supra-iliac SKT).

#### 3.3.2. Regression analysis.

Simple linear regression identified significant associations between several anthropometric measures and thinness, as defined by BMI-for-age indices. Specifically, thinness was associated with a mid-upper arm circumference (MUAC) of less than 21 cm (β = -0.38), skinfold thickness (S4SKT) below 25 cm (β = -0.34), hip circumference under 82 cm (β = -0.33), and waist circumference less than 64 cm (β = -0.21) (Table 4).

**Table 4:** Anthropometric measurements associated with thinness among ALHIV on ART follow-up.

Variable	Thinness (undernutrition with BMI-for-Age Z-score indicators)	
	Simple LR	Adjusted LR
	β coefficient (95%CI)	β coefficient (95% CI)
MUAC < 21 cm	-0.38 (-0.42, -0.26) ***	-0.37 (-0.42, -0.26) ***
(S4SKT < 25 cm	-0.34 (-0.37, -0.21) ***	-0.25 (-0.29, -0.13) ***
Hip circumference < 82 cm	-0.33 (-0.37, -0.20) ***	-0.16 (-0.23, -0.05) **
Waist circumference < 64 cm	-0.21 (-0.26, -0.10) ***	
Significant at *P value ≤ 0.05, **P value ≤ 0.01 and p*** value ≤ 0.001		

Model fitness –

F-statistics – 35.09, PV < 0.001 Adjusted R^2^ = 0.211; SE = 0.381; Durbin Watson = 1.935.

Model 1 - Predictors MUAC

Model 2 -Predictors: MUAC, S4SKT

Model 3 -Predictors: MUAC, S4SKT, and hip circumference

Dependent Variable: Thinnes growth

Multivariable stepwise backward regression confirmed these associations. A MUAC < 21 cm was independently associated with lower BMI-for-age Z-scores (β = -0.37, 95% CI: -0.42 to -0.26). A S4SKT < 25 cm was also significantly associated with thinness (β = -0.25, 95% CI: -0.29 to -0.13). Additionally, hip circumference < 82 cm was linked to a β of -0.16 (95% CI: -0.23 to -0.06).

These findings highlight the value of MUAC, skinfold thickness, and hip circumference as practical anthropometric indicators for identifying ALHIV at risk of malnutrition, reinforcing the need for routine monitoring and targeted nutritional interventions.

### 3.4. Healthcare professionals’ nutritional assessment, counselling, and support practices

#### 3.4.1. Nutritional assessment practices.

Most healthcare professionals (HCWs) reported using multiple methods to assess the nutritional status of ALHIV. Commonly utilized methods included anthropometric measurements (100%, n = 44), clinical assessments (93.3%, n = 41), and biochemical assessments (77.3%, n = 34). However, dietary intake assessments and food security evaluations were less commonly conducted, reported by only 36.4% (n = 16) and 27.3% (n = 12) of participants, respectively. Only one-quarter (25%, n = 11) reported using all four assessment methods (Table 5a).

**Table 5:** (a) Nutritional assessment practices reported by clinic healthcare workers for attendees at the ART clinics of selected hospitals in two regions of Ethiopia, 2024. (b) Nutritional counselling and education practices reported by clinic healthcare workers for attendees at the ART clinics of selected hospitals in two regions of Ethiopia, 2024. (c) Nutritional management and care/support practices of the study participants in selected hospitals of two regions, Ethiopia, 2024 (n = 44).

(a) Nutritional assessment practices reported by clinic healthcare workers for attendees at the ART clinics of selected hospitals in two regions of Ethiopia, 2024		
Variables	Description	Frequency N (%)
Anthropometric measurements used in ART clinics	Height	44 (100)
	Weight	44 (100)
	Body Mass Index	42 (95.5)
	Mid-upper Arm Circumference	25 (56.8)
	Skinfold Thickness	0 (0.0)
	Waist Circumference	3 (6.8)
	Waist-to-hip ratio	2 (4.5)
	Lean body mass	0 (0.0)
	Body Fat	0 (0.0)
	Grip strength	0 (0.0)
Biochemical Assessments undertaken for ALHIV in ART clinics	Glucose Test	32 (72.7)
	Blood Urea and Nitrogen test	33 (75.0)
	Creatinine Test	32 (72.7)
	BUN to Creatinine Ratio	16 (36.4)
	Calcium	4 (9.1)
	Total Protein	3 (6.8)
	Albumin	20 (45.5)
	Alkaline Phosphatase	21(47.7)
	Alanine Aminotransferase	22 (50.0)
	White Blood Cell count	35 (79.5)
	Red Blood Cell count	39 (88.6)
	Hemoglobin	39 (88.6)
	Hematocrit	39 (88.6)
	Mean corpuscular Volume	38 (86.4)
	Mean Corpuscular Hemoglobin	38 (86.4)
	Platelet count	25 (56.8)
	Helminth Infection (hookworm and ascaris)	17 (38.6)
Clinical Assessment conducted at the ART clinic	Bilateral pitting oedema	40 (90.9)
	Visible wasting	41 (93.2)
	Recent weight loss	41 (93.2)
	Dermatosis	18 (40.9)
	Eye signs (Bitot spot, corneal cloudiness, conjunctivitis, corneal ulceration)	17 (38.6)
	Palms, mucus membranes, and nail bed loss	35 (79.5)
(b) Nutritional counselling and education practices reported by clinic healthcare workers for attendees at the ART clinics of selected hospitals in two regions of Ethiopia, 2024		
**Variables**		**Frequency** **N (%)**
Availability of nutrition counselling and education for ALHIV at ART clinic (n = 44)		17 (38.6)
Regular practice of nutritional counselling and education to ALHIV during ART follow-up (n = 17)		10 (58.8)
Use GALIDRAA approaches nutrition counselling and education for ALHIV (n = 17)		4 (23.5)
Use ORPA approaches during discussion time for nutrition counselling and education for ALHIV (n = 17)		2 (11.8)
(c) Nutritional management and care/support practices of the study participants in selected hospitals of two regions, Ethiopia, 2024 (n = 44)		
**Variables**	**Description**	**Frequency** **N (%)**
Has standard admission criteria for nutrition care and support	Yes	21 (47.7)
Types of standard admission criteria used (n = 21)	Anthropometric Measurement (i.e., only BMI < 18.5 kg/m^2^)	17 (80.9)
	Combined Method (i.e., Anthropometric* and Clinical findings**)	4 (19.1)
Nutrition screening	Yes	36 (81.8)
Nutrition Case Management	Yes	36 (81.8)
Nutrition Supplementation	Yes	32 (72.7)
Type of supplementation provided [multiple responses] (n = 32)	Ready to use Therapeutic Food (RUTF)	32 (100)
	Ready to use Supplementary Food (RUSF)	5 (15.6)
	Others***	2 (6.25)
Supplementation delivery period (n = 32)	Every 2 weeks	5 (15.6)
	Every month	27 (84.4)
Availability of a strategy to detect sharing supplementation, e.g., with a sibling (n = 32)	Yes	13(40.6)
Strategies used to detect sharing (n = 13)	Progress Assessment	3 (23.1)
	Weight change/gain assessment	10 (76.9)
Has standard discharge criteria for nutrition care and support program	Yes	16 (36.4)

ALHIV – Adolescent Living with HIV; GALIDRAA –Greet, Ask, Listen, Identify, Discuss, Recommend, Agree, and Appoint; ORPA –Observe, Reflect, Personalize, and Act approaches during discussion time for nutrition counselling for ALHIV.

N.B: *Anthropometric measurements like MUAC<16 cm, BMI < 18.5 kg/m^2^, Weight-for-height Z-Score < -2 or -3

**Clinical signs like Visible severe wasting, recent weight loss, Bilateral pitting Oedema

***Others – Often supplementation of Oil, Teff flour, and Wheat flour.

All HCWs reported access to standardized weight and height measurement tools. However, only 63.6% (n = 28) used MUAC tapes, 9.1% (n = 4) had access to waist circumference tapes, and just 4.5% (n = 2) reported using skinfold calipers. No participants had access to more advanced tools such as dual-energy X-ray absorptiometry (DXA) or grip strength dynamometers.

Weight-for-age, height-for-age, and weight-for-height indices were routinely assessed by all HCWs. Most also reported regular evaluation of BMI (95.5%, n = 42), and more than half assessed MUAC (56.8%, n = 25). However, few assessed waist circumference or waist-to-hip ratio. None conducted comprehensive body composition assessments (e.g., skinfold thickness, lean body mass, or grip strength).

While biochemical testing was reported as routine practice, specific markers such as total protein and calcium were not frequently assessed. Although visual inspection for signs of malnutrition was commonly performed, less than half of HCWs routinely assessed for dermatosis or ocular signs of micronutrient deficiency (Table 5a).

#### 3.4.2. Counselling and nutrition education.

Only 38.6% (n = 17) of HCWs reported providing nutrition counselling and education during routine clinic visits, and among these, just over half (58.8%, n = 10) offered these services regularly. The WHO-endorsed GALIDRAA(Greet, Ask, Listen, Identify, Discuss, Recommend, Agree, Appoint) counselling framework was used by 23.5% (n = 4), while only 11.8% (n = 2) implemented the ORPA (Observe, Reflect, Personalize, Act) strategy during the discussion phase of counselling (Table 5b).

#### 3.4.3. Nutrition care and support services.

Approximately half of the participants (47.7%, n = 21) reported the presence of admission criteria for nutrition care services within their clinics, while only 36.4% (n = 16) indicated the presence of discharge criteria. Most clinics used BMI < 18.5 kg/m² as the sole admission threshold for nutritional care (81%, n = 17).

Nutrition screening (81.8%, n = 36), case management (81.8%, n = 36), and supplementation (72.7%, n = 32) were reported by the majority of respondents. All undernourished ALHIV were provided with ready-to-use therapeutic food (RUTF), though only 15.6% (n = 5) received ready-to-use supplementary food (RUSF). Most healthcare professionals (84%, n = 27) provided monthly supplementation; however, only 40.6% had strategies in place to monitor or prevent the sharing of supplements among siblings (Table 5c).

#### 3.4.4. Implementation support.

Most participants reported limited institutional support for nutritional care in ART clinics. Key gaps included the absence of standard operating procedures (SOPs), national nutrition guidelines, educational materials, and monitoring or reporting tools. Few participants had access to staff training, or on-site treatment services for undernourished ALHIV. Nonetheless, more than half (54.5%, n = 24) reported access to external referral systems for additional support (Table 6).

**Table 6:** Resources and activities to support staff in providing nutritional care and support for ALHIV on ART follow-up (n = 44).

Variables	Frequency N (%)
Nutrition care and support training available for ALHIV for ART staff	5 (11.4)
Standard operating procedures/ standards of practice (SOP) available for nutritional care	3 (6.8)
Nutrition support guidelines provided by the hospital	4 (9.1)
Job aids available, such as – algorithms, anthropometric reference cards, brochures, posters, pictures	3 (6.8)
Patient information and education resources available	3 (6.8)
Treatment for undernourished patients	8 (18.2)
Referral and linkage system available to support ALHIV	24 (54.5)
Monitoring checklist/multi-chart/card/ audit tools available	0 (0.0)
Reporting format and system to regional health bureau and others for monitoring purposes	9 (20.5)

#### 3.4.5. Healthcare professionals’ perspectives.

Over half of the respondents (54.5%, n = 24) expressed dissatisfaction with current nutrition assessment, counselling, and care practices for ALHIV. One participant noted:

*“Nutrition assessment, counselling, care, and support services for adolescents living with HIV are inadequate. This perception stems from a lack of integration of nutrition services and insufficient training for ART clinic staff on HIV and nutrition.”*

Despite routine reporting of anthropometric assessments, only 36.4% (n = 16) consistently conducted all three key measurements: weight, height, and BMI. Moreover, just 13.6% (n = 6) reported conducting effective clinical assessments, such as dietary intake evaluation or identifying clinical signs of malnutrition.

A respondent highlighted the complexity of nutritional assessment in practice:

*“Nutritional assessments are crucial, but limited methods are relied upon in practice. Given the complexity of malnutrition, accurately determining nutritional status is challenging.”*

Only 11.4% (n = 5) reported that nutrition supplementation and monitoring services were effectively delivered, and even fewer (4.5%, n = 2) noted effective delivery of psychosocial support.

Overall, 34% (n = 15) of healthcare professionals were dissatisfied with the quality of nutrition services at their facilities, citing the lack of integration with HIV care, incomplete screening protocols, insufficient training, and the absence of consistent nutritional support programs.

## 4. Discussion

This study reveals a critical public health concern: high levels of malnutrition among adolescents living with HIV (ALHIV) in Ethiopia. Utilizing multiple anthropometric indicators—BMI-for-age (BAZ), height-for-age (HAZ), and mid-upper arm circumference (MUAC)—we found alarmingly high rates of thinness, stunting, and acute malnutrition, particularly among mid-adolescents and male participants [30,31]. These findings underscore the urgent need for targeted, age- and gender-specific nutritional interventions.

The observed prevalence of malnutrition aligns with studies from Uganda and southern Ethiopia [15,32], yet exceeds rates reported in southwest Nigeria [33] and other regions of Uganda [34]. Such variation may reflect differences in socioeconomic status, dietary practices, healthcare access, and the availability of nutrition-focused services. The underutilization of structured and evidence-based nutritional interventions further compounds these disparities.

Socioeconomic vulnerability likely played a central role in shaping the nutritional outcomes observed among ALHIV in this study. Adolescents from households with limited income, food insecurity, and reduced caregiver support appeared to be disproportionately affected by acute malnutrition. In low-resource settings such as Ethiopia, HIV-affected households often experience compounded economic strain due to access to adequate and diversified diets [9,35]. Consistent with prior evidence, these findings suggest that undernutrition among ALHIV was not solely biologically mediated by HIV infection but also socially patterned by structural inequalities that limited food availability, dietary quality, and continuity of nutritional care [1,3,7,36].

Despite regular basic anthropometric measurements in ART clinics, the absence of more sensitive assessments—such as skinfold thickness, waist circumference, and DXA—limits healthcare providers’ ability to detect early or subclinical malnutrition. This shortfall likely contributes to the high burden of malnutrition observed in this cohort.

Furthermore, our findings reveal a substantial gap in nutrition counselling and education. While a minority of healthcare workers provide nutrition education, the limited use of structured tools such as GALIDRAA and ORPA indicates a lack of training and institutional support. These frameworks are crucial for delivering personalized, high-quality care, and their absence is likely to reduce the effectiveness of counselling practices.

The high burden of malnutrition observed in this cohort also appeared to be partially attributable to systemic weaknesses in healthcare delivery within routine HIV services. Nutritional assessment, counselling, and management were not consistently embedded into standard adolescent HIV care, and documentation of anthropometric indicators was often incomplete, placing adolescents at risk of undetected acute or chronic malnutrition and reduced opportunities for early nutritional intervention [37–41]. Similar challenges have been reported in other low-resource settings, where HIV programs prioritized virological and immunological monitoring over comprehensive nutritional care integrations [5,8,10,42].

Although most healthcare providers conduct nutrition screening and provide RUTF to severely malnourished ALHIV, many report inadequate access to assessment tools, treatment guidelines, and support services. Consistent with previous research [38,43], this lack of resources and training undermines the ability of clinics to deliver integrated, sustained nutritional care. The infrequent use of ready-to-use supplementary foods (RUSF) for moderate malnutrition and the lack of psychosocial support services further highlight systemic gaps in care delivery.

Healthcare workers’ perspectives reinforce these findings. Over half expressed dissatisfaction with existing nutrition services, citing poor integration with HIV care, absence of comprehensive screening, and limited access to tools and training. These concerns reflect an urgent need for investment in standardized protocols, staff development, and sustainable service delivery.

The heightened vulnerability of mid-adolescent males to malnutrition highlights the importance of age- and sex-specific nutritional interventions. While more than half of healthcare workers had access to referral and linkage systems, better integration of nutrition service into ART care, along with improved capacity building, is essential to improving outcomes. Addressing these issues requires a multi-faceted response including training, guideline development, structured assessment protocols, and consistent supplementation strategies.

### 4.1. Implications of the study

This study offers several important implications for strengthening nutritional care for adolescents living with HIV (ALHIV) in Ethiopia. In terms of clinical practice, healthcare providers should adopt more sensitive and diverse nutritional assessment techniques, which requires targeted training and improved access to appropriate equipment. Regarding counselling and education, evidence-based approaches such as GALIDRAA and ORPA should be integrated into routine care, accompanied by ongoing education and mentorship for healthcare workers to ensure effective implementation. From a public health and policy perspective, addressing socioeconomic barriers to nutrition is essential, and this can be achieved through community-based food security initiatives. Additionally, national HIV policies should incorporate standardized nutritional protocols, supported by adequate funding and logistical infrastructure. Finally, future research should focus on identifying effective, context-specific nutritional interventions for ALHIV,

### 4.2. Limitations

This study has several limitations. Self-reported data from ALHIV and healthcare providers may be affected by recall and social desirability bias, despite efforts to minimize these through tool validation and triangulation. The small sample size, particularly among healthcare workers, limits the generalizability of the findings and may not capture the full range of perspectives. Inconsistencies in data collector training could have influenced measurement accuracy, and some dietary recall data may be incomplete. The cross-sectional design prevents causal inferences, and external factors such as socio-political instability and regional differences were not controlled. Future research should use larger, more diverse samples, objective nutrition indicators, and longitudinal designs to strengthen the evidence on nutritional support for ALHIV.

## 5. Conclusion and recommendations

This study identified a substantial burden of undernutrition among adolescents living with HIV in Ethiopia, with approximately one-fifth of the participants classified as having severe acute malnutrition based on MUAC-for-age criteria and male adolescents significantly more likely to be acutely malnourished than their female counterparts. These findings underscore both the magnitude of severe nutritional vulnerability and the presence of important sex disparities in nutritional risk within this population.

The results highlight important gaps or inconsistencies in nutritional assessment, counselling, and support practices within ART clinics, reflecting systemic weaknesses in the integration of nutrition into routine HIV care. Despite regular clinical follow-up, many adolescents did not receive standardized nutritional screening, structured counselling, or timely referrals for nutritional support, indicating missed opportunities for early identification and management of malnutrition.

Together, these findings emphasize the urgent need to strengthen comprehensive integrated HIV and nutritional services through standardized assessment tools, clear clinical guidelines, targeted training for healthcare providers, and context-sensitive interventions that address both biological vulnerability and the underlying socioeconomic determinants of malnutrition. Improving the quality and consistency of nutritional care within ART programs is essential to enhance treatment outcomes, reduce morbidity, and improve the long-term health and wellbeing of adolescents living with HIV in Ethiopia.

## Supporting information

S1 Text Clinical measurements protocol. (DOCX)

S2 Text Operational definitions used in the study. (DOCX)

S1 Checklist Inclusivity in global research. (DOCX)
