# Supplementary material for: Nutritional status and gaps in nutritional care among adolescents living with HIV in Ethiopia: A multi-facility study
Source: PLOS Glob Public Health. 2026 Jul 8;6(7):e0004995. doi: 10.1371/journal.pgph.0004995 (PMC13345415; doi:10.1371/journal.pgph.0004995)
Supplement: S1 Text — (DOCX) [file pgph.0004995.s001.docx]

| **Supporting Information**  **S1 Text: Clinical Measurements Protocol** |
| --- |
| Clinical measurements adhered to standardized protocols to ensure consistency:   1. Participants removed heavy clothing before weight measurement. 2. Digital scales were calibrated and set to zero prior to each measurement, recording weight to the nearest 0.1 kg. 3. Height was measured with participants standing straight, and BMI was calculated (kg/m²). 4. Circumferences (arm, waist, hip) were measured using a non-fabric tape. 5. Skinfold thickness was assessed using Harpenden calipers at standardized sites to estimate body fat percentage. 6. Body composition was measured with a handheld body fat analyzer using bioelectrical impedance analysis (BIA). 7. Hand grip strength was assessed using a dynamometer, recording the highest squeeze from both hands. |
