# Supplementary material for: Nutritional status and gaps in nutritional care among adolescents living with HIV in Ethiopia: A multi-facility study
Source: PLOS Glob Public Health. 2026 Jul 8;6(7):e0004995. doi: 10.1371/journal.pgph.0004995 (PMC13345415; doi:10.1371/journal.pgph.0004995)
Supplement: S2 Text — (DOCX) [file pgph.0004995.s002.docx]

| **S2 Text: Operational definition** **used in the study** |
| --- |
| **Body Mass Index (BMI):** BMI is calculated as weight in kilograms divided by height in meters squared (Weight (kg) / height (m²)). **Categories:**   - Underweight: < 18.5 kg/m² - Normal weight: 18.5–24.9 kg/m² - Overweight: 25–29.9 kg/m² - Obesity: ≥ 30 kg/m² ^[^[^26^](#_ENREF_26)^,^ [^42^](#_ENREF_42)^]^   **Malnutrition:** Includes undernutrition, micronutrient deficiencies, and overweight/obesity, assessed using various indices tailored for different age groups.   - **For ALHIV aged 10–14 years:**   - Severe acute malnutrition: MUAC < 16 cm   - Moderate: MUAC 16–18.49 cm   - Normal: MUAC ≥ 18.5 cm - **For ages 15–19:**   - Severe: MUAC < 18.5 cm   - Moderate: MUAC 18.5–21 cm   - Normal: MUAC ≥ 21 cm^[^[^29^](#_ENREF_29)^]^.   **Nutritional Assessment:** A comprehensive evaluation to determine nutrient needs and nutritional status through medical history, dietary history, physical examination, anthropometric measurements, laboratory tests, and lifestyle data. It includes four categories: anthropometric, biochemical, clinical, and dietary intake^[^[^19^](#_ENREF_19)^,^ [^37^](#_ENREF_37)^,^ [^38^](#_ENREF_38)^]^:   - **Categories:**   - **Anthropometric:** Weight, height, BMI, MUAC, skinfold thickness, body fat percentage.   - **Biochemical:** Serum analysis of macro and micronutrient levels (e.g., hemoglobin, vitamins).   - **Clinical:** Signs of infection or malnutrition (e.g., low energy, opportunistic infections).   - **Dietary Intake:** Assessed through 24-hour recall and food diaries.   **Nutrition assessment, counselling, and management practice:** the provision of an integrated nutrition intervention for ALHIV during ART follow-up by health professionals working in ART clinics.   - **Integrated Nutrition Intervention for ALHIV includes:** - Systematic evaluation of nutritional status during ART follow-up. - Interactive dialogue for dietary problem management using GALIDRAA (greet, ask, listen, identify, discuss, recommend, agree, appoint) and ORPA (Observe, Reflect, Personalize, Act) approaches. - Providing nutrition care and support (e.g., management of acute malnutrition, nutrition supplementation, follow-up, setting practice standards).   **Nutritional Status** is defined as the condition influenced by diet, nutrient levels, and the balance between nutrient intake and requirements^[^[^33^](#_ENREF_33)^,^ [^37^](#_ENREF_37)^]^.  **Stunting** is identified by a height-for-age Z-score below -2 SD, with severe stunting below -3 SD^[^[^26^](#_ENREF_26)^,^ [^28^](#_ENREF_28)^,^ [^42^](#_ENREF_42)^,^ [^43^](#_ENREF_43)^]^.  **Thinness** is defined by a BMI-for-age Z-score below -2 SD, with severe thinness below -3 SD^[^[^26^](#_ENREF_26)^,^ [^28^](#_ENREF_28)^]^. |
