# Supplementary material for: Nutritional status and gaps in nutritional care among adolescents living with HIV in Ethiopia: A multi-facility study
Source: PLOS Glob Public Health. 2026 Jul 8;6(7):e0004995. doi: 10.1371/journal.pgph.0004995 (PMC13345415; doi:10.1371/journal.pgph.0004995)
Supplement: S1 Checklist — (DOCX) [file pgph.0004995.s003.docx]

Inclusivity in global research

PLOS’ policy on inclusivity in global research aims to improve transparency in the reporting of research performed outside of researchers’ own country or community and ensures that PLOS publications reporting global research adhere to high standards for research ethics and authorship. Authors of relevant research articles may be asked to complete the questionnaire below, which outlines ethical, cultural, and scientific considerations specific to inclusivity in global research. This questionnaire may be requested when researchers have travelled to a different country to conduct research, if research uses samples collected in another country, research with Indigenous populations or their lands, or if research is on cultural artefacts. Researchers travelling to another country solely to use laboratory equipment will not normally be required to complete the questionnaire. However, the questionnaire can be requested at the journal’s discretion for any submission – if you have been requested to complete this questionnaire by the PLOS journal you submitted to, please do so.

Please complete the questionnaire below and include this as a Supporting Information file with your manuscript. Note that if your paper is accepted for publication, this checklist will be published with your article in the supporting information files. Please ensure that you reference the checklist in the main body of your manuscript. We suggest adding a subsection ‘Inclusivity in global research’ to your Methods section and adding the following sentence: “Additional information regarding the ethical, cultural, and scientific considerations specific to inclusivity in global research is included in the Supporting Information (SX Checklist)”

The questions have been designed to be applicable to a wide range of study types, and there are subsections for both human subjects research and non-human subjects research. If any of the questions are not relevant to your research please mark them as “N/A” as appropriate.

**Ethical considerations, permits and authorship**

*This section is applicable to all research types.*

Provide details as to who granted permissions and/or consent for the study to take place in the Methods section of your manuscript. This should include the names of **all** ethics boards, governmental organizations, community leaders or other bodies that provided approval for the study. If individuals provided approval refer to these people by their role or title but do not list their name(s).

Reported on page number: 2-9

Permissions and ethical approvals for the study were obtained from the following bodies:

- **Institutional Ethics Board:**
  - Hawassa University College of Medicine and Health Sciences, Institutional Review Board (IRB), Ethiopia — Approval number: IRB/321/15
  - University of Technology Sydney (UTS), Human Research Ethics Committee (HREC), Australia — Approval number: ETH23-7873.
- **Governmental/Regional Health Authorities:** Relevant regional health offices overseeing the participating health facilities provided formal permission for the study to take place.
- **Health Facility Administrators:** Administrators at each participating health facility granted approval to conduct the study within their institutions and to access patient data.

As this study was conducted within health facilities, community-level permissions were not required. All individual participant consent procedures are described in detail in the Methods section of the manuscript.

Written informed consent and assent were obtained from all participants and/or their caregivers.

If there were any deviations from the study protocol after approval was obtained please provide details of these changes in the Methods section of your manuscript.
Did this study involve local collaborators that are residents of the country where the research was conducted or members of the community studied? If you do not have any authors from said communities, please provide an explanation for this below.

**Details:**
No deviations from the approved protocol occurred during data collection.

Reported on page number: 2-9

The study involved one local collaborator from Hawassa University and data collectors recruited from the participating health facilities. Their involvement ensured the research was culturally appropriate, feasible, and relevant to adolescents on antiretroviral therapy. All manuscript authors include researchers affiliated with Ethiopian institutions, reflecting meaningful local collaboration.

Everyone listed as an author should meet PLOS’ criteria for authorship and all individuals who meet these criteria should be included in the author byline, rather than the acknowledgements. For further information please see the journal’s Authorship Policy.

All individuals listed as authors on the manuscript meet PLOS’ criteria for authorship. Each author made substantial contributions to the conception, design, data collection, analysis, or interpretation of the study, and all have reviewed and approved the final manuscript. Individuals who contributed to the study but do not meet the authorship criteria are acknowledged appropriately in the Acknowledgements section.

**Human subjects research (e.g. health research, medical research, cross-cultural psychology)**

Did you obtain written informed consent from a representative of the local community or region before the research took place? How did you establish who speaks for the community? Details of written informed consent obtained from study participants should be reported separately in the Methods section of your manuscript.

Yes, written informed consent was obtained from a representative of the local community before the research commenced. To identify an appropriate representative, we consulted local leaders, health facility administrators, and community elders to determine who could legitimately speak on behalf of the community. This representative was provided with full information about the study’s objectives, procedures, risks, and benefits, and their written consent was obtained prior to starting the research.

Separately, written informed consent was also obtained from all study participants, as detailed in the Methods section of the manuscript, following local language (Amharic) explanations and the use of plain-language consent documents. Trained data collectors ensured that participants understood the study and had the opportunity to ask questions before consenting.

How did members of the local community provide input on the aims of the research investigation, its methodology, and its anticipated outcome(s)?

Although the study was conducted within health facilities, input from the local community was sought to ensure the research was relevant and appropriate. Consultations were held with health facility staff, adolescent patient representatives, and community health workers who interact with adolescents on antiretroviral therapy (ART). Their feedback helped refine the study aims, ensured that the data collection methods were culturally and contextually appropriate, and informed the anticipated outcomes in ways that would be meaningful for the community. This approach ensured that the study remained responsive to the needs and perspectives of the affected population while maintaining scientific rigor.

When engaging with the local community, how did you ensure that the informed consent documents and other materials could be understood by local stakeholders?

Will the findings of the research be made available in an understandable format to stakeholders in the community where the study was conducted (e.g. via a presentation, summary report, copies of publications, etc.)? Please provide details of how this will be achieved.

Although the study has been completed, the findings have not yet been disseminated to the community. A dissemination plan is in place to ensure the results are shared in an understandable and accessible format. The research team will prepare **plain-language summaries** of the findings (in Amharic and English) and distribute them through the participating health facilities. We will also hold **feedback and dissemination sessions** with adolescents, caregivers, and healthcare providers at the study sites. To ensure accessibility, findings will be presented using **simple language and visual aids**, such as infographics and short summary briefs. Copies of the final publications, accompanied by plain-language summaries, will be provided to local health offices, facility administrators, and other stakeholders.

**Non-human subjects research using specimens/ animals collected as part of the study, or those housed in archival collections. Examples include archaeology, paleontology, botany and zoology.**

Did the permission you obtained from a local authority to perform the study include an agreement on access to outputs and benefit sharing? This may include procedures to enable fair distribution of the benefits and resources arising from the research performed. Please include any details of Prior Informed Consent and Benefit Sharing Agreements obtained. These may be required by field-specific regulations, for example the Convention on Biological Diversity (CBD) and the associated Nagoya Protocol.

This study did **not include non-human subjects**. It involved human participants only — adolescents on antiretroviral therapy. All procedures, including informed consent and ethical approval, were conducted in accordance with human research ethics standards.

If the material used in your study was imported, please A) provide the year it was imported and B) indicate whether permits were obtained to import/export the materials used, C) provide details of any permits obtained. If this information is not available, please indicate this.

Not applicable. No materials were imported for this study, and therefore no import/export permits were required or obtained. The research involved human participants only, and all data were collected locally within the participating health facilities.

If you used archival specimens, please state how the material used in your study was acquired by the institute it is held in and provide details of any permits obtained for the original excavations/ sample collection. If this information is not available, please indicate this.

Not applicable. This study did not use archival specimens, and no materials from previous collections or excavations were involved. All data were collected directly from human participants within the participating health facilities.

How was the potential cultural significance of the materials collected in your study to local communities considered in your research design? Were Indigenous peoples and/or local researchers and institutions involved with archaeological excavations / collection of specimens? If so, please provide a description of their involvement.

Not applicable. No archaeological specimens or materials were collected in this study. The research focused exclusively on human participants (adolescents on antiretroviral therapy). However, local cultural considerations were respected through engagement with health facility staff, adolescent patient representatives, and community health workers, ensuring that study aims, methodology, and outcomes were culturally appropriate and relevant. Local researchers and staff were actively involved in the study design, data collection, and community engagement processes.

If your manuscript includes photographs of human remains please indicate whether authors obtained permission from descendants or affiliated cultural communities to do so.

Not applicable. This study did not include photographs of human remains. All data were collected from living human participants (adolescents on antiretroviral therapy).
