# Supplementary material for: Nutritional status and gaps in nutritional care among adolescents living with HIV in Ethiopia: A multi-facility study
Source: PLOS Glob Public Health. 2026 Jul 8;6(7):e0004995. doi: 10.1371/journal.pgph.0004995 (PMC13345415; doi:10.1371/journal.pgph.0004995)
Supplement: Attachment — Submitted filename: Response to Reviewers.docx [file pgph.0004995.s005.docx]

**Response to Reviewers**

**Manuscript ID:** PGPH-D-25-01852
**Title:** Critical Gaps in Nutritional Care for Adolescents Living with HIV: A Multi-Facility Study from Ethiopia

| **Comment No.** | **Reviewer/Editor Comment** | **Author Response** | **Location in Manuscript** |
| --- | --- | --- | --- |
| 1 | The study objectives are not distinctively outlined. Clearly stating the primary and secondary objectives would enhance the clarity and focus of the paper. | We revised the Introduction to explicitly state the primary and secondary objectives. The primary objective is assessing anthropometric and nutritional status of adolescents living with HIV, while the secondary objective is exploring healthcare professionals’ nutritional assessment, counselling, and support practices. | Introduction, page 3, lines 90–98 |
| 2 | Inconsistency in study duration: Section 2.1 lists August 2023–December 2023, Section 2.3.1 lists September 2023–February 2024. | The study period was corrected throughout the Methods section. The consistent study duration is **September 2023 to February 2024**. | Methods: Sections 2.1 & 2.3.1 |
| 3 | Discussion could be strengthened; include detailed discussion of healthcare delivery factors and socioeconomic determinants. | The Discussion was expanded to include analysis of healthcare delivery gaps, underutilization of nutritional counselling, and the influence of socioeconomic factors (household income, food insecurity, caregiver support) on malnutrition outcomes. | Discussion, Section 4, paragraphs 2–4 |
| 4 | Conclusion could be stronger by highlighting study-specific findings, e.g., severe acute malnutrition prevalence and sex differences. | The Conclusion was revised to emphasize key findings: ~20% of participants had severe acute malnutrition, and males were more likely to be acutely malnourished than females. We also linked these findings to recommendations for integrated nutritional care. | Conclusion and Recommendations, page 29–30 |
| 5 | Ensure data availability aligns with PLOS policies and provide repository details. | A Data Availability statement was added: *“The data supporting the findings of this study are available upon reasonable request. Access may be restricted to ensure participant confidentiality and compliance with ethical guidelines.”* All summary data are included in the manuscript and supporting files. | Data Availability, page 31 |
| 6 | Figures and tables: review for clarity and consistency. | All tables (1–6) and figures (1–3) were carefully reviewed. Labels, legends, and data presentation were checked and clarified where needed. No further changes were required. | Tables 1–6, Figures 1–3 |
| 7 | Include a complete PLOS inclusivity questionnaire. | Completed PLOS inclusivity questionnaire is attached as Supporting Information (Supplementary File). | Supporting Information |
| 8 | Submit revised manuscript with track changes and clean version. | Both files prepared: *“Revised Manuscript with Track Changes”* and *“Manuscript”* (clean version). | Submission package |
