# Supplementary material for: Nutritional status and gaps in nutritional care among adolescents living with HIV in Ethiopia: A multi-facility study
Source: PLOS Glob Public Health. 2026 Jul 8;6(7):e0004995. doi: 10.1371/journal.pgph.0004995 (PMC13345415; doi:10.1371/journal.pgph.0004995)
Supplement: Attachment — Submitted filename: Response to Editors and Reviewers.docx [file pgph.0004995.s006.docx]

**Response to Editors and Reviewers**

Manuscript ID: **PGPH-D-25-01852R1**
Title: **Nutritional Status and Critical Gaps in Nutritional Care Among Adolescents Living with HIV in Ethiopia: A Multi-Facility Study**

Dear Editor and Reviewers,

We sincerely thank the editor and reviewers for their constructive comments and suggestions. We have carefully revised the manuscript to address all comments. All changes have been incorporated into the revised manuscript and highlighted in the tracked-changes version.

Below we provide a detailed, point-by-point response to each comment.

**Comments and Responses**

| **Comment** | **Response** | **Location in Revised Manuscript** |
| --- | --- | --- |
| **1. Align the title with the main results in the abstract. While the title focuses on nutrition care gaps, the abstract includes burden of malnutrition.** | Thank you for this valuable suggestion. The title and abstract were revised to better reflect both the **nutritional status of adolescents living with HIV and gaps in nutritional care practices** within ART clinics. | Title page; Page 1 Line 1-3 |
| **2. Abstract – Methods: How were quantitative and qualitative data analysed?** | The abstract was revised to clarify the analytical approach. It now states that **quantitative data were analysed using descriptive statistics and regression analysis, while qualitative responses were analysed using thematic analysis.** | Abstract – Methods  Page 2. Line 22-34 |
| **3. Abstract – Results: What were the demographic characteristics of the study participants?** | Key demographic characteristics of the study participants were added to the abstract, including the **mean age of participants and sex distribution**. | Abstract – Results; Page 2, line 38-42 |
| **4. Abstract – Conclusion: Clarify statements about gaps in nutrition services.** | The conclusion was revised to more accurately reflect the results and avoid overstatement. The revised wording emphasizes **inconsistent implementation of nutritional care components within ART clinics rather than implying a universal absence of services**. | Abstract – Conclusion; page 2-3, Line 43-52 |
| **5. Include line numbering to facilitate proper review.** | Line numbering has been added throughout the revised manuscript to facilitate the review process. | Entire manuscript |
| **6. Introduction: What is the dual burden?** | We clarified the meaning of **“dual burden”** in the introduction, defining it as the coexistence of **undernutrition and emerging forms of malnutrition such as overweight and diet-related non-communicable diseases** among adolescents living with HIV. | Introduction; page 5; Line 88-91 |
| **7. What is the rationale for including the proportion of adolescents in the population?** | The rationale was clarified by explaining that adolescents represent **a large demographic group in Ethiopia**, making them a key target population for health and nutrition interventions. | Introduction; Page 5, Line 91-93 |
| **8. Provide references for the description of the study setting.** | References describing the Ethiopian HIV care system and ART service context were added to support the description of the study setting. | Methods – Study Setting, Page 6, Line 133-135 |
| **9. The sample size of healthcare workers was not explained.** | We clarified that all healthcare professionals directly involved in providing ART services to adolescents in the selected facilities were invited to participate. The final number of participants reflects the available staff and consented participants. | Methods – Sample Size Determination; page 7, Line 162-166 |
| **10. Provide a list of signs of malnutrition assessed during physical examinations.** | The description of clinical assessments was expanded to include specific signs assessed during physical examination, including **visible wasting, bilateral pitting oedema, palmar pallor, hair changes, and muscle loss.** | Methods – Data Collection; Page 8-9, Line 195-207 |
| **11. Rewrite methodology in active rather than passive voice.** | The methods section was revised to improve clarity and readability by using active voice where appropriate (e.g., “we conducted analyses using SPSS”). | Methods; Page 6-10 |
| **12. Justify the use of both Kolmogorov–Smirnov and Shapiro–Wilk tests for normality.** | A justification has been added explaining that **Shapiro–Wilk was used due to its higher statistical power for smaller samples**, while the **Kolmogorov–Smirnov test served as an additional verification method**, supported by visual inspection using Q-Q plots. | Methods – Data Analysis; page 9-10, Line 221- 241 |
| **13. Clarify the mixed methods design.** | The study design section was revised to explicitly state that the study used a **mixed-methods sequential explanatory design**, integrating quantitative findings with qualitative insights from healthcare professionals. | Methods – Study Design; page 6-7, Line 137-143 |
| **14. Ensure consistency in font types throughout the manuscript.** | The manuscript formatting was reviewed and standardized to ensure consistent font style and formatting. | Entire manuscript |
| **15. Define ORPA and GALIDRAA at first use.** | The full forms of **GALIDRAA (Greet, Ask, Listen, Identify, Discuss, Recommend, Agree, Appoint)** and **ORPA (Observe, Reflect, Personalize, Act)** were defined at their first appearance in the manuscript. | Results section; Page 14, Line 341-344 |

**Additional Changes Made**

In addition to addressing the editor’s comments, we made the following improvements:

- Clarified several methodological descriptions.
- Improved grammar and readability throughout the manuscript.
- Strengthened the discussion to better link findings with existing literature.
- Standardized terminology related to nutritional assessment and care practices.

**Files Submitted**

The following files have been submitted with this revision:

1. **Response to Reviewers**
2. **Revised Manuscript with Track Changes**
3. **Clean Manuscript (without tracked changes)**
4. **Completed PLOS Inclusivity in Global Research Questionnaire**

We appreciate the editor’s and reviewers’ thoughtful feedback and believe the revisions have significantly strengthened the manuscript.

Thank you for considering our revised submission.

Sincerely,

**Dr. Meless Gebrie Bore**
