# Supplementary material for: Nutritional status and gaps in nutritional care among adolescents living with HIV in Ethiopia: A multi-facility study
Source: PLOS Glob Public Health. 2026 Jul 8;6(7):e0004995. doi: 10.1371/journal.pgph.0004995 (PMC13345415; doi:10.1371/journal.pgph.0004995)
Supplement: Peer review history

# Peer review: Decision Letter 0

**DOI:** 10.1371/journal.pgph.0004995.r001

**Authors:** Jennifer Tucker

8 Jan 2026

PGPH-D-25-01852

Critical Gaps in Nutritional Care for Adolescents Living with HIV: A Multi-Facility Study from Ethiopia

PLOS Global Public Health

Dear Dr. Bore,

Thank you for submitting your manuscript to PLOS Global Public Health. After careful consideration, we feel that it has merit but does not fully meet PLOS Global Public Health’s publication criteria as it currently stands. Therefore, we invite you to submit a revised version of the manuscript that addresses the points raised during the review process.

Please note that we have only been able to secure a single reviewer to assess your manuscript. We are issuing a decision on your manuscript at this point to prevent further delays in the evaluation of your manuscript. Please be aware that the editor who handles your revised manuscript might find it necessary to invite additional reviewers to assess this work once the revised manuscript is submitted. However, we will aim to proceed on the basis of this single review if possible.

Please submit your revised manuscript by Feb 22 2026 11:59PM. If you will need more time than this to complete your revisions, please reply to this message or contact the journal office at globalpubhealth@plos.org. When you're ready to submit your revision, log on to https://www.editorialmanager.com/pgph/ and select the 'Submissions Needing Revision' folder to locate your manuscript file.

Please include the following items when submitting your revised manuscript:

- A letter that responds to each point raised by the editor and reviewer(s). You should upload this letter as a separate file labeled 'Response to Reviewers'.
- A marked-up copy of your manuscript that highlights changes made to the original version. You should upload this as a separate file labeled 'Revised Manuscript with Track Changes'.
- An unmarked version of your revised paper without tracked changes. You should upload this as a separate file labeled 'Manuscript'.

Guidelines for resubmitting your figure files are available below the reviewer comments at the end of this letter.

We look forward to receiving your revised manuscript.

Kind regards,

Jennifer Tucker, PhD

Staff Editor

PLOS Global Public Health

Journal Requirements:

1. Please include a complete copy of PLOS’ questionnaire on inclusivity in global research in your revised manuscript. Our policy for research in this area aims to improve transparency in the reporting of research performed outside of researchers’ own country or community. The policy applies to researchers who have travelled to a different country to conduct research, research with Indigenous populations or their lands, and research on cultural artefacts. The questionnaire can also be requested at the journal’s discretion for any other submissions, even if these conditions are not met. Please find more information on the policy and a link to download a blank copy of the questionnaire here: https://journals.plos.org/globalpublichealth/s/best-practices-in-research-reporting. Please upload a completed version of your questionnaire as Supporting Information when you resubmit your manuscript.

If the reviewer comments include a recommendation to cite specific previously published works, please review and evaluate these publications to determine whether they are relevant and should be cited. There is no requirement to cite these works unless the editor has indicated otherwise.

Additional Editor Comments (if provided):

[Note: HTML markup is below. Please do not edit.]

Reviewers' comments:

Reviewer's Responses to Questions

**Comments to the Author**

1. Does this manuscript meet PLOS Global Public Health’s publication criteria? Is the manuscript technically sound, and do the data support the conclusions? The manuscript must describe methodologically and ethically rigorous research with conclusions that are appropriately drawn based on the data presented.

Reviewer #1: Yes

**********

2. Has the statistical analysis been performed appropriately and rigorously?

Reviewer #1: Yes

**********

3. Have the authors made all data underlying the findings in their manuscript fully available (please refer to the Data Availability Statement at the start of the manuscript PDF file)?

The PLOS Data policy requires authors to make all data underlying the findings described in their manuscript fully available without restriction, with rare exception. The data should be provided as part of the manuscript or its supporting information, or deposited to a public repository. For example, in addition to summary statistics, the data points behind means, medians and variance measures should be available. If there are restrictions on publicly sharing data—e.g. participant privacy or use of data from a third party—those must be specified.

Reviewer #1: Yes

**********

4. Is the manuscript presented in an intelligible fashion and written in standard English?

PLOS Global Public Health does not copyedit accepted manuscripts, so the language in submitted articles must be clear, correct, and unambiguous. Any typographical or grammatical errors should be corrected at revision, so please note any specific errors here.

Reviewer #1: Yes

**********

5. Review Comments to the Author

Please use the space provided to explain your answers to the questions above. You may also include additional comments for the author, including concerns about dual publication, research ethics, or publication ethics. (Please upload your review as an attachment if it exceeds 20,000 characters)

Reviewer #1: The manuscript addresses an important and probably neglected area in the adolescent and HIV care. The study findings carry strong implications for strengthening integrated HIV and nutrition programs across Ethiopia.

The study is scientifically sound, with a clear focus on anthropometric and nutritional status among adolescents living with HIV (ALHIV) and identifying critical gaps in nutritional care practices among healthcare providers. However, the study objectives are not distinctively outlined. Clearly stating the primary and secondary objectives would enhance the clarity and focus of the paper.

The methodology is well described and appropriate for the research question. The sampling methods used seem justified. However, there is an inconsistency in the reported study duration - section 2.1 lists the study period as August 2023 to December 2023, where as section 2.3.1 mentions patient recruitment from September 2023 to February 2024, which could be reporting error, that needs to be clarified.

The data analysis section is robust, the authors have effectively assessed normality, correlation, regression analysis. The inclusion of both quantitative and qualitative variable added depth to the findings.

The results are comprehensive and logically presented, particularly the BMI for age, MUAC and skin fold thickness. The findings provide insight over at risk adolescents for malnutrition. The overall data presentation in the various tables, figures was well organized and effectively supports the text, enhancing the readability of the manuscript.

The Discussion although relevant, could be further strengthened, the authors referenced several related studies, and could use their findings to justify the findings of the current study. More detailed discussion of certain variables like healthcare delivery factors, or the socio economic factors could enrich the interpretation of the study.

The conclusion is focussed on the broader implications of improving nutritional care among adolescents living with HIV. However, it would appear more stronger if highlighted on the study specific findings like about 1/5th of the study participants can be categorized as severe acute malnutrition or male participants are more likely to be acutely malnourished than female participants - by highlighting key determinants identified through this study. This would emphasize with the broader recommendations that already mentioned in the manuscript.

Overall, the manuscript appears to conform to the STORBE guidelines for cross-sectional studies.

The authors are also encouraged to ensure the data availability in line with the PLOS data sharing policies and to provide repository details if applicable.

To summarize, this is a valuable and timely study addressing a key population like adolescents and their nutritional care within HIV individuals. With minor revisions for clarity, consistency and deeper discussion, the manuscript would be a informative contribution to the literature.

**********

6. PLOS authors have the option to publish the peer review history of their article (what does this mean?). If published, this will include your full peer review and any attached files.

**Do you want your identity to be public for this peer review?**  If you choose “no”, your identity will remain anonymous but your review may still be made public.

For information about this choice, including consent withdrawal, please see our Privacy Policy.

Reviewer #1: **Yes:** Upasana Raj Battina

**********

[NOTE: If reviewer comments were submitted as an attachment file, they will be attached to this email and accessible via the submission site. Please log into your account, locate the manuscript record, and check for the action link "View Attachments". If this link does not appear, there are no attachment files.]

-->Figure Resubmissions:

While revising your submission, we strongly recommend that you use PLOS’s NAAS tool (https://ngplosjournals.pagemajik.ai/artanalysis) to test your figure files. NAAS can convert your figure files to the TIFF file type and meet basic requirements (such as print size, resolution), or provide you with a report on issues that do not meet our requirements and that NAAS cannot fix.

After uploading your figures to PLOS’s NAAS tool - https://ngplosjournals.pagemajik.ai/artanalysis, NAAS will process the files provided and display the results in the "Uploaded Files" section of the page as the processing is complete. If the uploaded figures meet our requirements (or NAAS is able to fix the files to meet our requirements), the figure will be marked as "fixed" above. If NAAS is unable to fix the files, a red "failed" label will appear above. When NAAS has confirmed that the figure files meet our requirements, please download the file via the download option, and include these NAAS processed figure files when submitting your revised manuscript.

# Author comment: Author response to Decision Letter 1

**DOI:** 10.1371/journal.pgph.0004995.r002

25 Jan 2026

Attachment Submitted filename: Response to Reviewers.docx

# Editor report: Decision Letter 1

**DOI:** 10.1371/journal.pgph.0004995.r003

**Authors:** Jennifer Tucker, Andrew Kazibwe

5 Feb 2026

PGPH-D-25-01852R1

Critical Gaps in Nutritional Care for Adolescents Living with HIV: A Multi-Facility Study from Ethiopia

PLOS Global Public Health

Dear Dr. Bore,

Thank you for submitting your manuscript to PLOS Global Public Health. After careful consideration, we feel that it has merit but does not fully meet PLOS Global Public Health’s publication criteria as it currently stands. Therefore, we invite you to submit a revised version of the manuscript that addresses the points raised during the review process.

Thank you for addressing the previous round of comments. We encourage you to further revise the manuscript using the additional comments provided.

Please submit your revised manuscript by Mar 22 2026 11:59PM. If you will need more time than this to complete your revisions, please reply to this message or contact the journal office at globalpubhealth@plos.org. When you're ready to submit your revision, log on to https://www.editorialmanager.com/pgph/ and select the 'Submissions Needing Revision' folder to locate your manuscript file.

Please include the following items when submitting your revised manuscript:

- A letter that responds to each point raised by the editor and reviewer(s). You should upload this letter as a separate file labeled 'Response to Reviewers'.
- A marked-up copy of your manuscript that highlights changes made to the original version. You should upload this as a separate file labeled 'Revised Manuscript with Track Changes'.
- An unmarked version of your revised paper without tracked changes. You should upload this as a separate file labeled 'Manuscript'.

Guidelines for resubmitting your figure files are available below the reviewer comments at the end of this letter.

We look forward to receiving your revised manuscript.

Kind regards,

Andrew Kazibwe, MBChB, MMED

Academic Editor

PLOS Global Public Health

Journal Requirements:

1. Please include a complete copy of PLOS’ questionnaire on inclusivity in global research in your revised manuscript. Our policy for research in this area aims to improve transparency in the reporting of research performed outside of researchers’ own country or community. The policy applies to researchers who have travelled to a different country to conduct research, research with Indigenous populations or their lands, and research on cultural artefacts. The questionnaire can also be requested at the journal’s discretion for any other submissions, even if these conditions are not met. Please find more information on the policy and a link to download a blank copy of the questionnaire here: https://journals.plos.org/globalpublichealth/s/best-practices-in-research-reporting. Please upload a completed version of your questionnaire as Supporting Information when you resubmit your manuscript.

If the reviewer comments include a recommendation to cite specific previously published works, please review and evaluate these publications to determine whether they are relevant and should be cited. There is no requirement to cite these works unless the editor has indicated otherwise.

Additional Editor Comments (if provided):

Title: I suggest that the authors align the title with the main results in the abstract. While the title focusses on nutrition care gaps, the content of the abstract includes burden of malnutrition and associated factors.

Abstract – Methods: How were quantitative and qualitative data analysed?

Abstract - Results: What were the demographic characteristics of the study participants?

Abstract - Conclusion: The authors emphasise that substantial gaps exist in nutrition services, yet the wording of the results is not necessarily to this effect. A gap refers to a difference between the current reality and a desired goal/ideal. How terrible is it that only 38.6% of ALHIV assessed dietary intake (compare this to a program goal/target).

The authors should include line numbering to facilitate proper review

Introduction: What is the dual burden?

What is the rationale of including the proportion of adolescents in the total population?

What makes adolescents vulnerable to malnutrition?

The primary aim of the study was to assess the nutritional status of ALHIV, but this is not reflected in the abstract title. Please harmonise.

While the study title focusses on gaps in care, this was neither the primary nor secondary aim of the study. Please harmonise.

Please provide references for the description of the study setting provided in the methods section.

The authors present two components of a quantitative study: one involving ALHIV, and another involving healthcare workers. Yet there is no presentation of how the sample size of healthcare workers was determined. Please rectify.

Please share the sample allocation table by study site, as a supplementary file.

Provide a complete list of the signs of malnutrition that the physical examinations sought to find.

I suggest that the authors re-write the entire methodology section in active, rather than passive voice, as a good writing practice. For example, instead of, “all quantitative data were analysed using SPSS version 21”, this can be re-written as, “we conducted analyses of quantitative data using SPSS version 21”.

What was the rationale for using both the Kolmogorovi-Smirnov and Shapiro-Wilk tests for normality assessment? Why wasn’t a single test adequate? What was the outcome of this assessment?

It appears the authors used a mixed methods sequential explanatory approach to triangulation of quantitative and qualitative findings. This should be clearly stated and justified.

Please ensure consistency in font types throughout the manuscript.

The authors report quantitative findings from the healthcare worker interviews, yet the enrolment of health care workers in the study did not follow proper quantitative sampling methodologies.

Is the WHO-endorsed GALIDRAA counseling framework recommended for use by the Ethiopian policy guidelines?

Define ORPA and GALIDRAA at first use in the manuscript

[Note: HTML markup is below. Please do not edit.]

Reviewers' comments:

[NOTE: If reviewer comments were submitted as an attachment file, they will be attached to this email and accessible via the submission site. Please log into your account, locate the manuscript record, and check for the action link "View Attachments". If this link does not appear, there are no attachment files.]

-->Figure Resubmissions:

While revising your submission, we strongly recommend that you use PLOS’s NAAS tool (https://ngplosjournals.pagemajik.ai/artanalysis) to test your figure files. NAAS can convert your figure files to the TIFF file type and meet basic requirements (such as print size, resolution), or provide you with a report on issues that do not meet our requirements and that NAAS cannot fix.

After uploading your figures to PLOS’s NAAS tool - https://ngplosjournals.pagemajik.ai/artanalysis, NAAS will process the files provided and display the results in the "Uploaded Files" section of the page as the processing is complete. If the uploaded figures meet our requirements (or NAAS is able to fix the files to meet our requirements), the figure will be marked as "fixed" above. If NAAS is unable to fix the files, a red "failed" label will appear above. When NAAS has confirmed that the figure files meet our requirements, please download the file via the download option, and include these NAAS processed figure files when submitting your revised manuscript.

# Author comment: Author response to Decision Letter 2

**DOI:** 10.1371/journal.pgph.0004995.r004

5 Mar 2026

Attachment Submitted filename: Response to Editors and Reviewers.docx

# Editor report: Decision Letter 2

**DOI:** 10.1371/journal.pgph.0004995.r005

**Authors:** Jennifer Tucker, Andrew Kazibwe, Julia Robinson

28 May 2026

Nutritional Status and Gaps in Nutritional Care among Adolescents Living with HIV in Ethiopia: A Multi-Facility Study

PGPH-D-25-01852R2

Dear Dr. Bore,

We are pleased to inform you that your manuscript 'Nutritional Status and Gaps in Nutritional Care among Adolescents Living with HIV in Ethiopia: A Multi-Facility Study ' has been provisionally accepted for publication in PLOS Global Public Health.

Before your manuscript can be formally accepted you will need to complete some formatting changes, which you will receive in a follow up email. A member of our team will be in touch with a set of requests.

Please note that your manuscript will not be scheduled for publication until you have made the required changes, so a swift response is appreciated.

IMPORTANT: The editorial review process is now complete. PLOS will only permit corrections to spelling, formatting or significant scientific errors from this point onwards. Requests for major changes, or any which affect the scientific understanding of your work, will cause delays to the publication date of your manuscript.

If your institution or institutions have a press office, please notify them about your upcoming paper to help maximize its impact. If they'll be preparing press materials, please inform our press team as soon as possible -- no later than 48 hours after receiving the formal acceptance. Your manuscript will remain under strict press embargo until 2 pm Eastern Time on the date of publication. For more information, please contact globalpubhealth@plos.org.

Thank you again for supporting Open Access publishing; we are looking forward to publishing your work in PLOS Global Public Health.

Best regards,

Julia Robinson

Executive Editor

PLOS Global Public Health

***********************************************************

Reviewer Comments (if any, and for reference):
